# Prostaglandin E_2_ regulates senescence and post-senescence neoplastic escape in primary human keratinocytes

**DOI:** 10.18632/aging.206149

**Published:** 2024-11-18

**Authors:** Elise Srour, Nathalie Martin, Claire Drullion, Clémentine De Schutter, Joëlle Giroud, Adrien Pioger, Julie Deslé, Laure Saas, Joe Nassour, Julien Théry, Gauthier Decanter, Nicolas Penel, Chantal Vercamer, Clara Salazar-Cardozo, Corinne Abbadie, Olivier Pluquet

**Affiliations:** 1 CNRS, Inserm, CHU Lille, Institut Pasteur de Lille, UMR9020 – U1277 – CANTHER - Cancer Heterogeneity, Plasticity and Resistance to Therapies, University of Lille, Lille F-59000, France; 2 Direction of Clinical Research and Innovation, Oscar Lambret Center, Lille, France; 3 Department of Surgical, Oscar Lambret Center, Lille, France; 4 CHU Lille, ULR 2694 - Metrics: Evaluation des Technologies de Santé et des Pratiques Médicales, University of Lille, Lille, France; 5 Department of Biochemistry and Molecular Genetics, University of Colorado School of Medicine, Aurora, CO 80045, USA

**Keywords:** PTGS2, prostaglandins, EP receptors, senescence, neoplastic transformation, keratinocyte

## Abstract

Aging of the epidermis partially occurs as a consequence of epidermal cell senescence, a non-proliferative state in which cells remain metabolically active and acquire changes in their secretome. We previously reported that senescent normal human epidermal keratinocytes (NHEKs) have two opposite outcomes: either cell death by excess of autophagic activity or escape from senescence to give rise to post-senescence neoplastic emerging (PSNE) cells. In this study, we investigated the role of PTGS2, the inducible enzyme of the prostaglandin biosynthesis pathway, in the onset of NHEK senescence and in the switch from senescence to pre-transformation. We provide evidence that the PTGS2/PGE_2_/EP4 pathway plays a critical role in NHEK senescence as well as in senescence escape. We show that treating proliferating NHEKs with prostaglandin E_2_ (PGE_2_) or with an agonist of one of its receptors, EP4, induced the establishment of the senescent phenotype, according to several markers including the senescence-associated β-galactosidase activity. Conversely, treating already senescent NHEKs with an antagonist of EP4, or knocking-down PTGS2 by siRNA resulted in the decrease of the percentage of senescence-associated β-galactosidase-positive cells. We also demonstrate that the PSNE frequency was significantly decreased upon PTGS2 silencing by siRNA, pharmacological PTGS2 inhibition, or treatment by an EP4 antagonist, while on the contrary treatments with PGE_2_ or EP4 agonist increased the PSNE frequency. These results indicate that the PTGS2/PGE_2_/EP4 pathway is required to induce and maintain the senescent phenotype of NHEKs, and that PGE_2_ level is a potential determinant of the initial steps of the age-related oncogenic process.

## INTRODUCTION

The skin epidermis has a high rate of cell turnover to continuously reconstruct itself and to ensure the protection of the body. This protection can be compromised during physiological aging and photoaging due to UV overexposure, both conditions associated with skin inflammation and accumulation of senescent cells [1–4]. Keratinocyte senescence may impair epidermis functions leading to pathological conditions including the development of non-melanoma skin carcinoma which are the human cancer with the highest incidence [5].

Senescence is a cellular state known as an anti-tumor barrier, that can be induced by different stimuli including shortening of telomeres leading to replicative senescence (RS) or various endogenous or exogenous genotoxic agents leading to stress-induced premature senescence (SIPS) [6, 7]. The cell cycle arrest of senescent cells often depends on the activation of two main pathways, p53/p21^WAF1^ and/or p16^INK4^/pRb, in an interconnected or independent manner [8]. Regarding RS, high p21^WAF1^ expression was found during the final 2–3 passages before the onset of the senescent plateau, but its expression does not persist, suggesting that it is not essential for maintaining the senescence phenotype [9, 10]. p16^INK4A^ seems to take the relay, with an expression increased later [9, 10], but robustly maintained along the senescence plateau (see for review [11]). Regarding SIPS, the cell cycle arrest can be ensured through activation of the p53/p21^WAF1^ or the p16^INK4A^/Rb pathway, or through both (reviewed in [12–14]). Senescent cells display deep molecular changes, including alterations in metabolic, epigenetic, and gene expression programs [12, 14], leading to an increase in senescence-associated β-galactosidase (SA-β-Gal) activity and to profound changes in the senescence-associated secretory phenotype (SASP) which is enriched in pro-inflammatory cytokines, growth factors and remodeling enzymes of the extracellular matrix [7, 14–16]. Growing evidence showed that the SASP can play a critical role in tumorigenesis [17].

Epidermal keratinocytes display at senescence most of the characteristics of an adaptive response to stress [12]. Using a model of *in vitro* cultured Normal Human Epidermal Keratinocytes (NHEKs) and samples of human skin from donors at different ages, we have shown that senescence in epidermal keratinocytes occurs without any significant telomere shortening, but in response to an increase in intracellular concentration of reactive oxygen species (ROS). This oxidative stress leads to a cell cycle arrest mediated by the sole p16/Rb pathway. It also results in a high macroautophagic activity, associated with an increase in Beclin1 and a decrease in BCL2 expressions, leading to cell death without activation of the apoptotic pathway [18–20]. If most of senescent NHEKs in culture die by an excess of autophagy, a very few of them nevertheless remain alive and are permissive to the generation of neoplastic post-senescent cells [12]. Indeed, we have shown that a few rare senescent NHEKs can spontaneously and systematically escape from the senescent state. They re-enter cell cycle via a process we called PSNE (Post-Senescence Neoplastic Escape) which leads to clonal populations of daughter cells having acquired characteristics similar to those observed during the early stages of tumor initiation [21–23]. More importantly, when xenografted in *nude* mice, these PSNE cells developed in disseminated skin lesions such as hyperplasias and small non-melanoma skin carcinomas, providing evidence of their tumorigenic potential [22, 23].

The biosynthesis of prostaglandins (PGs) begins with the release of arachidonic acid from phospholipid membranes by the enzyme phospholipase A2. Arachidonic acid is then metabolized by the prostaglandin endoperoxide synthases PTGS1 and PTGS2 (also called cyclooxygenases COX1 and COX2) to form prostaglandin H_2_ (PGH_2_). PGH_2_ is the precursor of all series of prostaglandins and thromboxanes (TX). It is the substrate of different tissue-specifically expressed PG and TX synthases to generate a range of prostanoids including PGE_2_, PGD_2_, PGI_2_, PGF_2_α, and TXA_2_ [24]. Prostanoids exert their biological effects in a paracrine, autocrine, or intracrine manner through binding with prostanoid receptors. Prostanoid receptors control various biological functions not only through their sensitivity to the prostaglandins themselves, but also through the different receptor isoforms and the diversity of coupled G-proteins [25]. The active site of PTGSs is inhibited by most of non-steroidal anti-inflammatory drugs (NSAIDs). PTGS1 has been defined as a constitutive and ubiquitous enzyme that generates PGs controlling physiological homeostasis including platelet function and renal blood flow [26] [27]). In contrast, PTGS2 is an inducible enzyme whose expression is increased by various stimuli including growth factors, cytokines, and chemokines [26]. PTGS2 is involved in various processes leading to inflammation [28, 29], and plays a causal role in cancer [30, 31].

Several studies pointed the role of PTGS2 in senescence and aging of the dermis: (i) PTGS2 expression is increased in human aged skin, including fibroblasts of the dermis, compared to normal young skin [32, 33] and, in accordance, NS398, a selective PTGS2 inhibitor, has been shown to reduce skin aging in mice [34]; (ii) PTGS2 takes part in replicative senescence of *in vitro* cultured normal human dermal fibroblasts (NHDFs) [35], and NS398 also reduced this function [36]. The mechanisms were not fully dissected. We have shown that PGE_2_ acts on NHDF senescence via the pool of intracellular EP3 receptors [37]. Recently, PTGS2 was suggested to regulate the SASP composition through PGE_2_ [38].

Although PTGS enzymes were shown to contribute to the early stages of keratinocyte differentiation [39], nothing is known on the role of PTGSs in epidermis aging and keratinocyte senescence. We therefore investigated the functional involvement of the PTGS2 pathway in senescence of NHEKs and its impact on post-senescence neoplastic escape. By using functional approaches (including RNA interference, pharmacological inhibitors of PTGS2 activity, and agonists and antagonists of EPs), we identified the PTGS2/PGE_2_/EP4 pathway as a driver in both the establishment of senescence in NHEKs, and the promotion of the very initial phases of neoplastic transformation by senescence escape in a context of aging.

## RESULTS

### PTGS2 is upregulated in normal human epidermal keratinocytes during *in vitro* senescence and skin aging

The present study was performed using six different donors of NHEKs, from different sex, age and ethnicity group. As we already published [18–21, 40, 41], in standard culture conditions, these NHEKs enter senescence in 1 to 3 weeks and 10 to 15 cumulative population doublings according to the cell donor (Supplementary Figure 1A). At the senescence plateau, NHEKs display SA-β-Gal activity (Supplementary Figure 1B), cell spreading (Supplementary Figure 1C), and increase in cell size and granularity as observed by flow cytometry (Supplementary Figure 1D).

**Figure 1:**
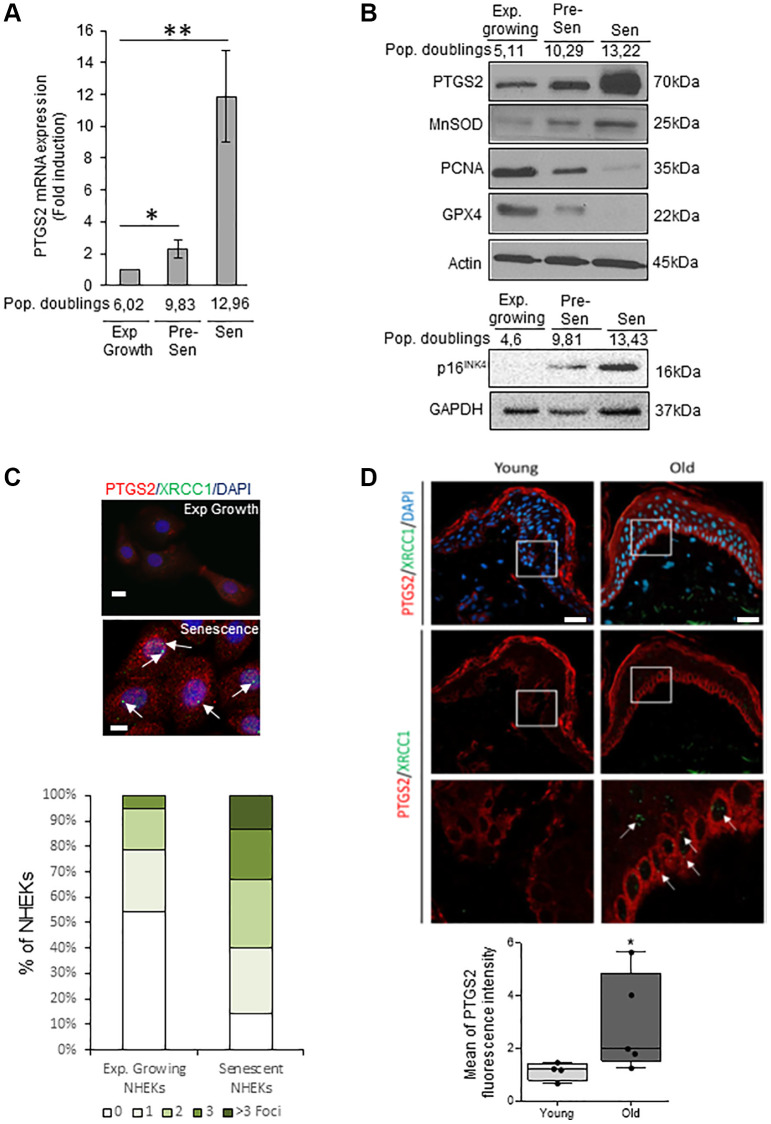
**PTGS2 is up-regulated during NHEK *in vitro* senescence and skin aging.** (**A**) The mRNA levels of PTGS2 were measured by RT-qPCR in extracts from exponentially growing, pre-senescent and senescent NHEKs (donor 4F0315). PTGS2 levels were normalized to EAR levels. The bars represent the mean ±SD (*p* < 0.05; ^**^p < 0.01). (**B**) PTGS2, MnSOD, PCNA, and GPX4 protein levels were evaluated by Western Blot in extracts from exponentially growing, pre-senescent and senescent NHEKs (upper panel: donor 4F0315; lower panel: donor K40FH1). The gel was equicharged with extracts from an equal number of cells. The equicharge was verified *a posteriori* by detecting the levels of actin and GAPDH. (**C**) Exponentially growing and senescent NHEKs (donor K3MC1) were fixed and processed for immunofluorescence detection of endogenous proteins PTGS2 (*red*) and XRCC1 (*green*). Cell nuclei were detected by DAPI staining (*blue*). Upper panel: Representative confocal photomicrographs of PTGS2 inmmunostaining and XRCC1 foci (white arrows). Bars represent 20 µm. Lower panel: XRCC1 foci were quantified. Measures were done in five independent microscopic fields for a total of at least 100 cells for each condition. The histogram represents the average ± S.D. of five counts. Results are representative of at least two independent experiments. (**D**) Immunofluorescence detection of PTGS2 (*red*) performed in sections of skin samples from human young (*n* = 4) and old (*n* = 5) healthy subjects (see Material and Method) (^*^p < 0.05). Cell nuclei were detected by DAPI staining (*blue*). Upper panel: representative confocal microscopy images for epidermis and dermis of a young (37 years old) and an elderly donor (85 years old). The squares delimit the below images at higher magnification. Bars represent 40 µm. Lower panel: scatter dot plots indicating the mean fluorescence intensity in cells of the basal layer. A minimum of 25 cells per sample were selected individually in order to obtain a mean fluorescence for each donor. The horizontal black lines denote median values and the boxes the interquartile ranges (IQR). Vertical lines extend from max value (upper quartile + 1.5*IQR) to min value (lower quartile – 1.5*IQR).

We investigated the PTGS2 expression at the mRNA and protein levels in exponentially growing, pre-senescent and senescent NHEKs. RT-qPCR analyses show that PTGS2 mRNA levels are increased at senescence compared to exponentially growth phase (Figure 1A). This up-regulation was confirmed by Western Blot (Figure 1B) and was correlated to an increase in the protein levels of p16^INK4a^, MnSOD, a marker of redox control that we previously showed as upregulated in senescent NHEKs [20], and to a decrease in PCNA, a marker of proliferation, and in GPX4, an antioxidant marker whose expression is down-regulated at senescence [42]. By immunofluorescence, the endogenous PTGS2 was barely detectable in growing NHEKs while it was highly expressed in 100% of senescent NHEKs identified by the presence of XRCC1 foci, as marker of DNA single-strand breaks (SSBs) (Figure 1C). To assess the *in vivo* relevance of these results, we analyzed PTGS2 protein levels in skin sections of young (18–40 years old) and old (>55 years old) healthy subjects by immunofluorescence. In skin sections of young people, PTGS2 protein levels were not detected in the dermis and hardly any in the epidermis. In skin section of old people, PTGS2 expression was significantly increased, mainly in the epidermis, in the basal layer containing proliferating keratinocytes, and in the upper layers containing terminally differentiated keratinocytes (Figure 1D). The staining intensity measure showed a statistically significant difference between the two groups (*P* < 0.01) (Figure 1D). These data are in agreement with PTGS2 immunohistological staining found in aged skin epidermis by Habib et al. [32] and confirm that an induction of PTGS2 expression is associated with senescence and aging of human epidermal keratinocytes.

### PTGS2 controls the establishment and maintenance of some senescence markers in NHEKs

**Figure 2:**
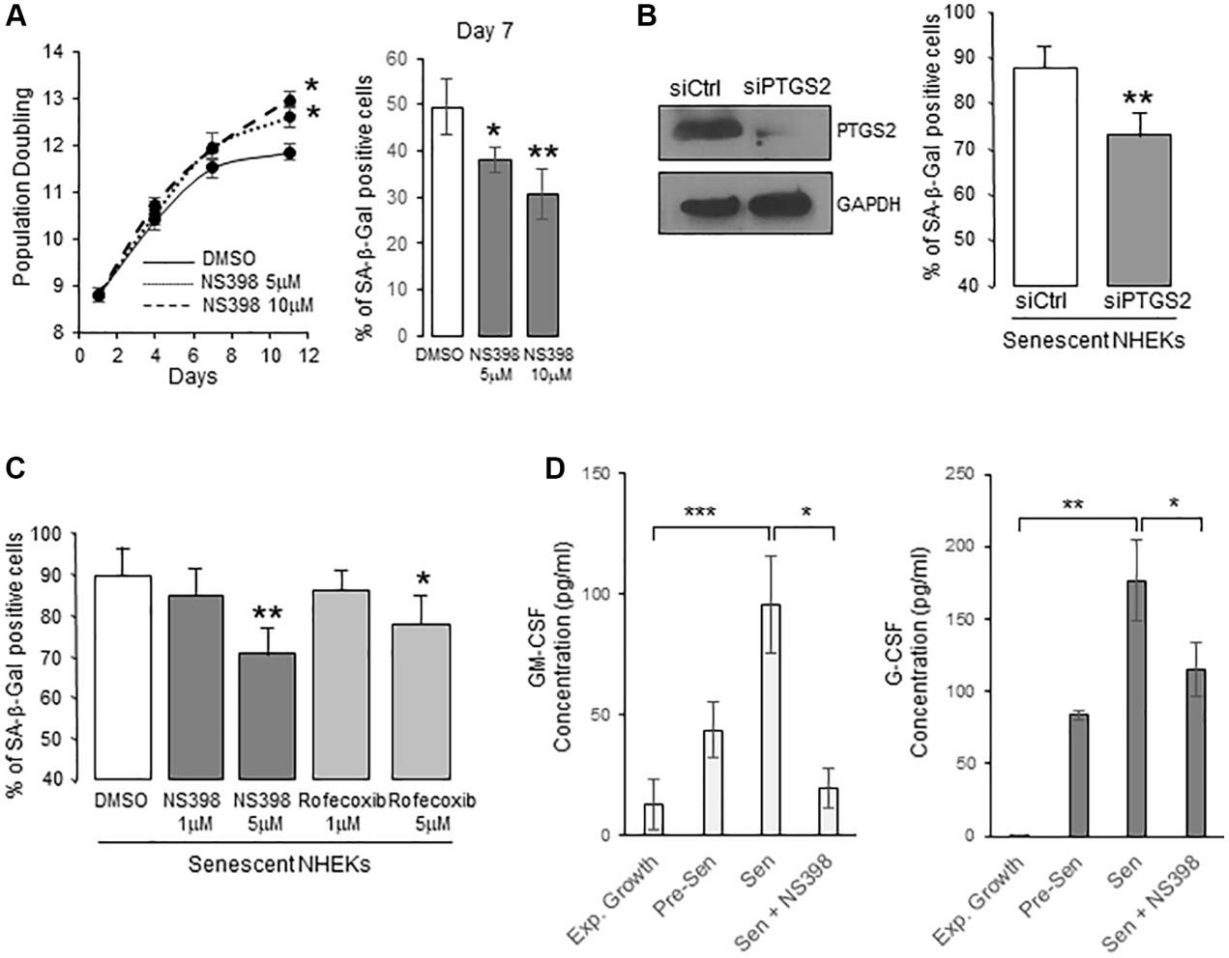
**PTGS2 induces and maintains NHEK senescence.** (**A**) NHEKs (donor 4F0315) were treated with NS398 at 5 or 10 µM or DMSO every 48 h. Left panel: During the treatment, cells were passaged when reaching 70% confluence, counted, and the number of population doublings was calculated (see Methods). The experiment was performed in triplicate, each point representing the mean of three counts. Significant differences between DMSO control and 5 or 10 µM NS398 treatment are indicated. Right panel: Senescent NHEKs were treated as in (**A**), then, a SA-β-Gal assay was performed seven days post-treatments. (**B**) Senescent NHEKs (donor K23FC1) were transfected with a pool of 4 siRNAs targeting PTGS2, or with non-target siRNAs. Left panel: Evaluation of the efficacy of the siRNAs by Western Blot. GAPDH was used as a loading control. Right panel: Four days after transfection, a SA-β-Gal assay was performed. The bars represent the mean ±SD of three counts of blue cells (^**^p < 0.01). (**C**) Senescent NHEKs (donor K23FC1) were treated with NS398 or rofecoxib at the indicated concentrations for four days. Then, a SA-β-Gal assay was performed. The bars represent the mean ±SD of three counts of blue cells (^*^p < 0.05; ^**^p < 0.01). (**D**) ELISA assays for measuring the amounts of GM-CSF and G-CSF in the conditioned media (secreted) of exponentially growing, pre-senescent and senescent NHEKs (donor K40FH1) treated or not with NS398 (5 µM) for 16 hrs. Measures were performed in triplicate. The bars represent the mean ± SD of three counts. Significant differences are indicated with asterisks with ^*^p < 0.05; ^**^p < 0.01; ^***^p < 0.001.

To determine whether PTGS2 is causally involved in the onset of senescence in NHEKs, we first cultured proliferating NHEKs (at 8.8 population doublings) in the presence of 5 or 10 µM NS398, an inhibitor of PTGS2 activity. We then followed the appearance of senescence criteria. After seven days, NS398 partly prevented the establishment of the senescence growth plateau, and the appearance of the SA-β-Gal activity (Figure 2A), suggesting that PTGS2 was necessary for the occurrence of NHEK senescence. We then silenced PTGS2 expression by RNA interference or inhibited its activity with two different pharmacological inhibitors (NS398 and rofecoxib) in already senescent NHEKs to assay whether PTGS2 contributes to the maintenance of senescence. The efficacy of PTGS2 silencing was checked by Western-blotting experiments which indicated that the expression of PTGS2 at the protein level was highly repressed after four days post-transfection (Figure 2B). At this same point, PTGS2 silencing significantly reduced the percentage of SA-β-Gal-positive cells (Figure 2B). Similar results were obtained with the highest concentrations of PTGS2 inhibitors (Figure 2C). We also examined the consequences of PTGS2 inhibition on the SASP. We first analysed the composition of conditioned media (CM) from proliferating and senescent NHEKs using a cytokine array. We identified different cytokines/chemokines that were significantly over-secreted by senescent NHEKs compared to proliferating ones, such as GM-CSF (=CSF2), G-CSF (=CSF3), IL10, CXCL1, CCL5 (Supplementary Figure 2). Surprisingly, IL-6, IL-8, or TNFα were not overdetected in senescent NHEKs. They are nevertheless considered as major components of the SASP, but most often established using models fibroblasts at replicative senescence, confirming that SASP composition is influenced by the cell-type and the senescence inducer [16]. We then examined whether the secretion of GM-CSF and G-CSF was dependent on PTGS2 activity. For that, we collected the CM from proliferating or senescent NHEKs exposed or not to NS398 and determined the concentration of GM-CSF and G-CSF by ELISA. The results indicated that PTGS2 activity was required for the secretion of GM-CSF and G-CSF (Figure 2D).

All together, these results suggest that PTGS2 is involved in the establishment and maintenance of senescence in NHEKs.

### PTGS2 does not contribute to NHEK senescence by producing an oxidative stress

In previous studies, we demonstrated that *in vitro* and *in vivo* senescence of human epidermal keratinocytes results from an increase in ROS concentration [18, 19, 40]. However, we still have not identified the origin of this oxidative stress. Since the PTGS2 catalytic activity of conversion of arachidonic aci into PGH_2_ generates a side-production of ROS, mainly O_2_^−^ and OH [43–45], we made the hypothesis that PTGS2 could contribute to NHEK senescence by this mechanism. We therefore first determined the level of ROS generated by the PTGS2 activity in NHEKs by silencing PTGS2 or by using the selective PTGS2 inhibitors NS398 and rofecoxib. ROS concentrations were measured by fluorimetry using H_2_DCFDA, a cell permeant probe which fluoresces upon oxidation. As already shown [19], the ROS levels highly increased in senescent NHEKs compared to proliferating ones. However, neither the pharmacological inhibition (Supplementary Figure 3A), nor the silencing of PTGS2 (Supplementary Figure 3B) altered the overall ROS production in proliferating or senescent NHEKs (compared to their respective controls). This indicates that the ROS-generating activity of PTGS2 does not significantly contribute to the overall ROS production involved in the induction and maintenance of NHEK senescence.

### PTGS2 contributes to NHEK senescence through the production of PGE_2_

**Figure 3:**
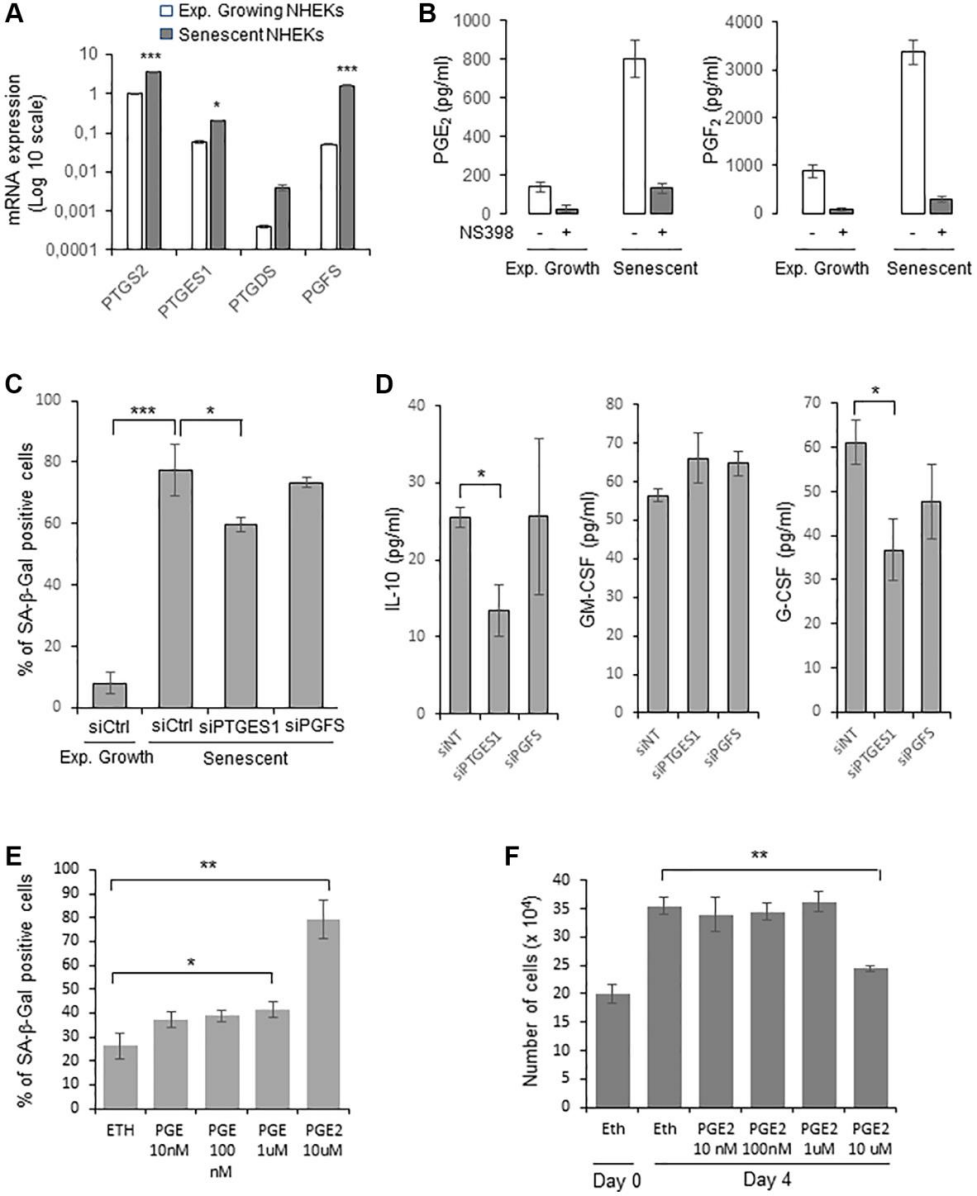
**PGE_2_ contributes to the establishment and maintenance of senescence in NHEKs.** (**A**) PTGS2, PTGES1, PTDGS, and PGFS mRNA levels were measured by RT-qPCR in NHEKs (donor K67FA1) at the exponential growth phase or at the senescence plateau and were normalized to EAR levels. Results are presented in log 10 scale relative to PTGS2 expression in exponentially growing NHEKs (^*^p < 0.05; ^***^p < 0.001). (**B**) The amount of PGE_2_ and PGF_2_ in the culture media (secreted) of exponentially growing or senescent NHEKs (donor K67FA1) were measured by a competitive assay (see Material and Methods). Measures were performed in triplicate. The bars represent means ± SD. (**C**) Senescent NHEKs (donor K40FH1) were transfected with a pool of 4 siRNAs targeting PTGES1 or PGFS, or with non-target siRNAs (siCtrl). Four days after transfection, a SA-β-Gal assay was performed. The bars represent the mean ± SD of three counts (^*^p < 0.05; ^***^p < 0.001). (**D**) The amounts of IL-10, GM-CSF and G-CSF in the conditioned media (secreted) were measured by ELISA assays in NHEKs (donor K40FH1) treated as in (**C**). Measures were performed in triplicate. The bars represent the mean ± SD. Significant differences are indicated with asterisks with ^*^p < 0.05. (**E**) Pre-senescent NHEKs (donor K23FC1) were treated or not with 10 nM to 10 µM PGE_2_ 4-times a day. The percentage of SA-β-Gal positive cells 4 day after the beginning of the treatment was determined. The bars represent the mean ± SD of three counts (^*^p < 0.05; ^**^p < 0.01). (**F**) Pre-senescent NHEKs (donor K23FC1) were treated as in (**E**), the number of cells was then determined. The bars represent the mean ± SD of three counts (^**^p < 0.01).

We then examined whether PTGE2 could control NHEK senescence through its PG production. The main PGs produced by normal human keratinocytes are PGE_2_, PGF_2_α and PGD_2_ [46]. We therefore first investigated by RTqPCR the gene expression level of PGE synthases (PGES) downstream of PTGSs: PTGES1 and PTGES2 (producing PGE_2_), PGFS (producing PGF2α), PGIS (producing PGI_2_), PTGDS (producing PGD_2_), and TXAS1 (producing TXA_2_), in both growing and senescent NHEKs. As expected for human keratinocytes [46], PGIS and TXAS1 expressions were not detected. Surprisingly, PTGES2 expression was not detected as well. Only the expression of PTGES1 and PGFS significantly increased at senescence compared to exponentially growth phase (Figure 3A). Based on these results, we measured the amount of PGE_2_, PGD_2_, and PGF_2_α secreted by growing and senescent NHEKs. Both the secretion of PGE_2_ and PGF_2_ increased in senescent compared to growing NHEKs. As controls, these elevated levels were abolished in the presence of NS398 (Figure 3B). PGD_2_ was not detected, in accordance with the very low level of PTGDS expression.

We therefore focussed on the investigation of the role of PGE_2_ and PGF_2_ on NHEK senescence. For that, we silenced PTGES1 and PGFS expression by RNA interference and, after having verified siRNA efficiencies (Supplementary Figure 4), we determined the effects on senescence markers. After 4 days post-transfection, only PTGES1 silencing significantly reduced the percentage of SA-β-Gal-positive cells (Figure 3C). We also collected the CM from senescent NHEKs treated or not with siRNA and determined by ELISA the levels of GM-CSF, G-CSF and IL-10. The results indicated that only PTGES1 was able to significantly modulate the secretion of IL-10 and G-CSF, but not of GM-CSF (Figure 3D). It is worth to note that adding exogenous PGE_2_ partially rescued IL-10 production in senescent NHEKs knocked-down for PTGSE1 (Supplementary Figure 5).

Then, to evaluate the potential role of PGE_2_ to induce NHEK senescence through an autocrine, paracrine or intracrine mechanism, we treated cultures of proliferating NHEKs with increasing concentrations of exogenous PGE_2_ and examined whether this could induce premature senescence. Given the poor stability of prostaglandins in aqueous solutions, we added PGE_2_ to the culture medium four times a day. We observed a statistically significant increase in the number of SA-β-Gal-positive cells at 1 and 10 µM of PGE_2_ (Figure 3E), as well as a decrease in cell proliferation at 10 µM of PGE_2_ (Figure 3F). All together, these data indicate that the PTGS2/PTGES1/PGE_2_ pathway is causally involved in the onset and maintenance of senescence in NHEKs.

### EP4 activation triggers senescence in NHEKs

**Figure 4:**
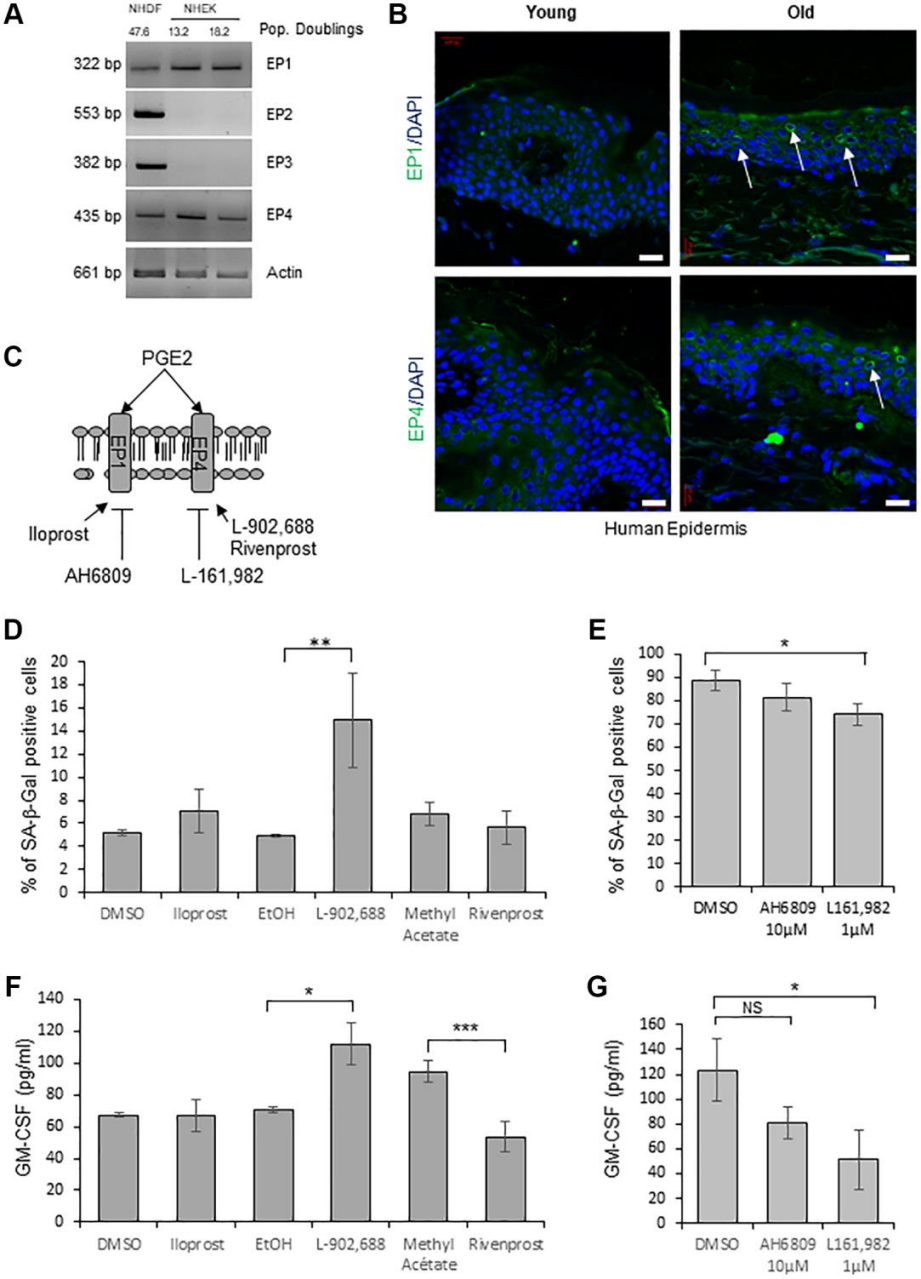
**EP4 receptors mediate senescence of epidermal keratinocytes.** (**A**) The mRNA levels of EPs were detected by RT-PCR in extracts from exponentially growing and senescent NHEKs (donor 4F0315) and in NHDFs at the replicative senescence plateau. Actin levels were used as control. The number of population doublings for NHDFs and NHEKs are indicated. (**B**) Immunofluorescence detection of EP1 and EP4 (*green*) performed in sections of skin samples from human young and old healthy subjects (see Material and Method). Cell nuclei were detected by DAPI staining (*blue*). Representative confocal microscopy images of the epidermis of a young (37 years old) and an elderly donor (83 years old). Positive staining of EP1 and EP4 (white arrows) are shown. Bars represent 20 µm. (**C**) Scheme of PGE_2_ receptors and their agonists/antagonists used in the following experiments. (**D**) NHEKs (donor K40FH1) at the exponential growth phase were treated with the EP1 or EP4 agonists (Iloprost, L-902,688 and Rivenprost, respectively at 100 ng/mL, 1 µM and 100 nM) for 4 days. The percentage of SA-β-Gal-positive cells was determined 4 days after the beginning of treatment. SA-β-Gal-positive cells were counted in at least 3 different microscopic fields. The bars represent the mean ± SD of at least 3 counts (^**^p < 0.01). (**E**) Senescent NHEKs (donor K23FC1) were treated with EP1 and EP4 antagonists (AH6809 and L-161,982, respectively at 10 µM and 1 µM). The percentage of SA-β-Gal-positive cells was determined 4 days after the beginning of treatment. SA-β-Gal-positive cells were counted in at least 3 different microscopic fields. The bars represent the mean ± SD of at least 3 counts (^*^p < 0.05). (**F**) NHEKs (donor K40FH1) at the exponential growth phase were treated as in (**D**), the amounts of GM-CSF in the conditioned media (secreted) were measured by an ELISA assay. Measures were performed in triplicate. The bars represent the mean ± SD. Significant differences are indicated with asterisks with ^*^p < 0.05 and ^***^p < 0.001. (**G**) NHEKs (donor K40FH1) at the exponential growth phase were treated as in (**E**), and the amount of GM-CSF in the conditioned media (secreted) was measured by an ELISA assay. Measures were performed in triplicate. The bars represent the mean ± SD. Significant differences are indicated with asterisks with ^*^p < 0.05.

In order to determine through the activation of which EP receptor PGE_2_ induces senescence in NHEKs, we first evaluated by RT-PCR the expression of the different EPs. Surprisingly, among the four EPs, we detected only EP1 and EP4 mRNAs in NHEKs, in contrast to NHDFs in which the four EPs were expressed (Figure 4A). We next analyzed EP1 and EP4 protein levels in skin sections of young and old healthy subjects. Immunofluorescence against EP1 and EP4 proteins showed that their expressions were significantly increased in the epidermis of old people compared to young ones (Figure 4B).

To test the involvement of a signalisation through EP1 and/or EP4 in the induction of NHEK senescence, we used specific cell-permeant pharmacological agonists/antagonists of each EP (Figure 4C). We first treated exponentially growing NHEKs with agonists of EP1 (Iloprost) and EP4 (L-902,688 or Rivenprost) and found that only one EP4 agonist (L-902,688) increased the number of SA-β-Gal-positive cells (Figure 4D). We then treated already senescent NHEKs with antagonists of EP1 (AH6809) and EP4 (L-161,982) [47] and found that antagonizing EP4 but not EP1 significantly decreased the number of SA-β-Gal-positive cells (Figure 4E). Similarly, only one EP4 agonist (L-902,688) led to an increased production of GM-CSF by proliferating NHEKs (Figure 4F) and only the EP4 antagonist to a decreased release of GM-CSF by already senescent NHEKs (Figure 4G).

These results strongly suggest that the PTGS2/PGE_2_/EP4 pathway is involved in the onset and the maintenance of NHEK senescence.

### The PTGS2/PGE_2_/EP4 pathway regulates the post-senescence neoplastic emergence frequency

**Figure 5:**
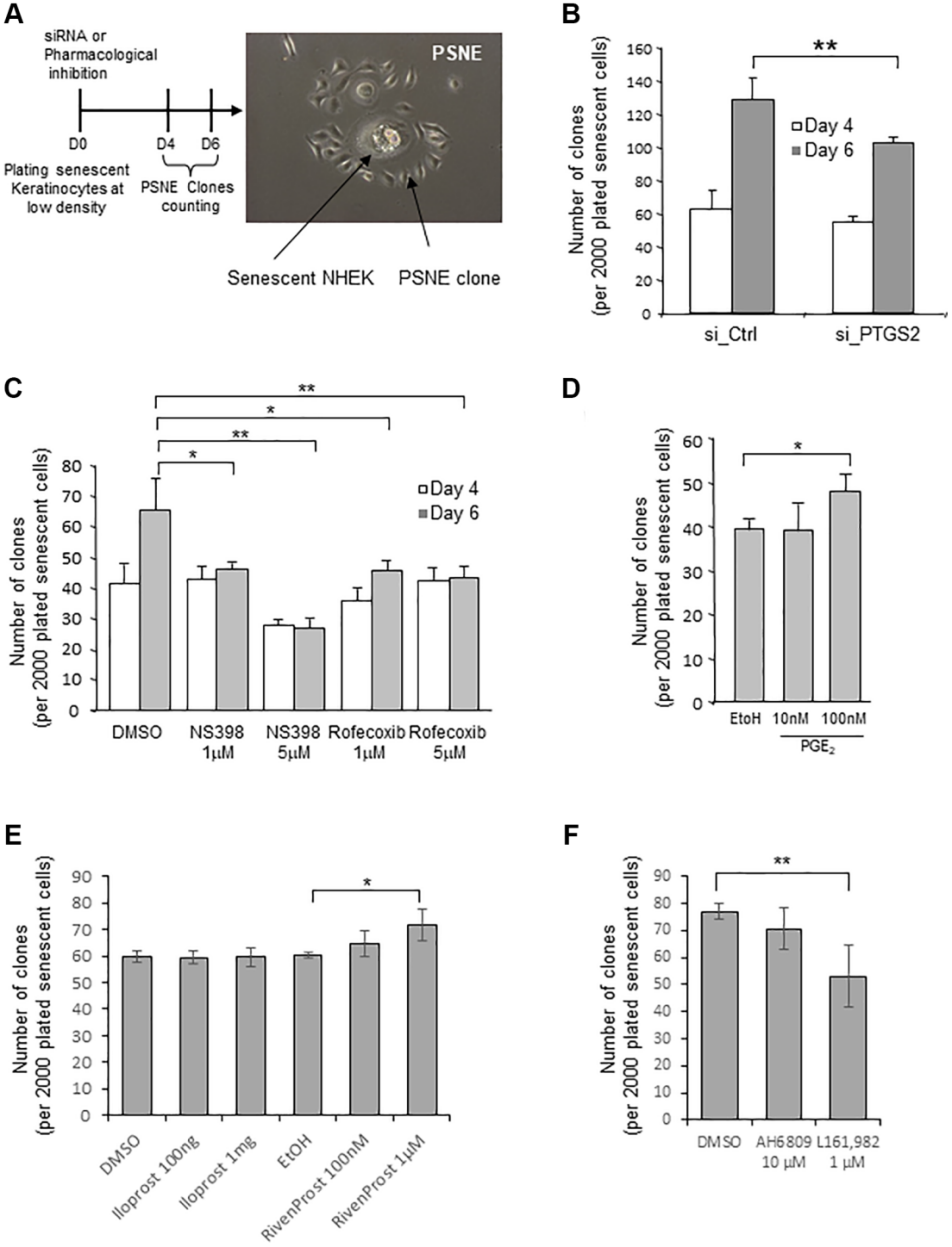
**Loss of PTGS2 activity reduces preneoplastic senescence escape of NHEKs.** (**A**) Left panel: Scheme depicting the experimental process. Right panel: representative phase contrast microscopy image of what was counted as a clone in the following experiments. (**B**) Senescent NHEKs (donor K67JA1) were subjected to PTGS2 siRNAs vs. non-target control siRNAs. After four- or six-days post-transfection, emerging clones were manually counted under microscopic examination after fixation and coloration with crystal violet. (**C**) Senescent NHEKs (donor K23C1) were subjected to PTGS2 pharmacological inhibition by NS398 or Rofecoxib at the indicated concentrations. Four- or six-days post-treatment, emerging clones were manually counted as in B. (**D**) Senescent NHEKs (donor KNBMC1) were treated or not with 10 or 100 nM PGE_2_ 4-times a day. After four days of treatment, the number of emerging clones was counted as in B. (**E**) Senescent NHEKs (donor K40FH1) were treated with the EP1 or EP4 agonists (Iloprost and Rivenprost at the indicated concentrations). After four days of treatment, the number of emerging clones was counted as in B. (**F**) Senescent NHEKs (donor K40FH1) were treated with EP1 and EP4 antagonists (AH6809 and L-161,982, respectively at 10 µM and 1 µM). After four days of treatment, the number of emerging clones was counted as in B. In (**B**–**F**), the bar chart represents the mean ± SD of the 3 independent measures (^*^p < 0.05; ^**^p < 0.01). In (**B**–**F**), each result is representative from 3 independent experiments, with each experiment corresponding of three or four technical replicates.

In previous studies, we had shown that a few senescent NHEKs can spontaneously and systematically escape from the senescent state and re-enter cell cycle at a frequency of 10^−2^ to 10^−4^ to generate a progeny of (pre)neoplastic cells by an unusual asymmetric mitosis mechanism (budding). We named this process “Post senescence neoplastic emergence” (PSNE) [22, 41] (Figure 5A and Supplementary Figure 6). Here, we investigated whether PTGS2 silencing or pharmacological inhibition could alter the process of PSNE. To this end, senescent NHEKs were transfected with siRNAs against PTGS2, plated at low density, and monitored for emergence during up to 6 days. The emergence frequency was quantified by counting the number of PSNE clones per plated senescent cells. Silencing PTGS2 significantly decreased the PSNE frequency (Figure 5B). Similarly, the treatment of senescent NHEKs by non-toxic concentrations of NS398 or rofecoxib significantly decreased the PSNE frequency in a dose-dependent manner (Figure 5C). No cell death was observed in the remaining adherent keratinocytes after siRNA or pharmacological treatments (not shown). Then we wondered whether, on the contrary, an excess of PGE_2_ given to senescent NHEKs could favour or not neoplastic evasion. To address this question, senescent NHEKs plated at low density were treated with exogenous PGE_2_ (10 and 100 nM) and the emergence frequency was monitored as above. The results show that 100 nM PGE_2_ in the culture medium of senescent NHEKs increased the PSNE frequency (Figure 5D). Next, we determined whether the PSNE frequency can be modified in response to EP1/4 activity modulation. We first treated senescent NHEKs with the EP4 agonist L-902,688, but it turned out to be toxic after 4 days of treatment (which was not the case in proliferating NHEKs, see Figure 4D), preventing the determination of the emergence frequency. So, we replaced L-902,688 with another EP4 agonist called Rivenprost, and showed that only Rivenprost, but not the EP1 agonist Iloprost, increased the emergence frequency (Figure 5E). We then wanted to confirm these results by treating senescent NHEKs with the EP1 and EP4 antagonists. Only the EP4 antagonist L-161,982, but not the EP1 antagonist AH6809, treatment significantly reduced the PSNE frequency (Figure 5F).

All these results indicate that the neoplastic senescence escape was controlled, at least in part, by the PTGS2/PGE_2_/EP4 pathway.

## DISCUSSION

Our data indicate that the PTGS2 level and the production of PGE_2_ are increased during *in vitro* senescence of NHEKs, as well as during aging of skin epidermal keratinocytes. This hormone production plays a role both in the establishment of the senescence state, in its maintenance, as well as in the escape to generate (pre)neoplastic cells.

We had demonstrated in several previous studies [19] [20] that senescence in epidermal keratinocytes is mainly the consequence of an increase of an endogenous oxidative stress whose origin remained unestablished. By producing ROS [43–45], the catalytic activity of PTGS2 could have been one of these sources of oxidative stress. However, our results demonstrate that it is not the case, since silencing or pharmacologically inhibiting PTGS2 did induce a partial reversal of senescence but without altering total ROS levels. We therefore explored whether PTGES2 contributes to senescence directly through the prostanoids produced by the downstream prostaglandin synthases. Although PGE_2_ and PGF_2_ were both highly overproduced by senescent NHEKs in consequence of an up-regulation of PTGES1 and PGFS respectively, our results indicate that PGE_2_ was the main protanglandin involved in the induction and maintenance of the senescent phenotype, and in senescence escape. PGE_2_ mainly acts paracrinally, autocrinally or intracrinally by binding to membrane or intracellular forms of the G protein-coupled receptors EP1, 2, 3 and 4 [48]. Our results indicate that only EP1 and EP4 are expressed in epidermal keratinocytes. Although both of them are slightly up-regulated with *in vitro* senescence or during skin aging, EP4 seems to play the most important role in NHEK senescence induced by PGE_2_. Indeed, inhibiting EP4 using a pharmacological antagonist partly reversed some senescence markers, whereas activating EP4 with an agonist induced a premature senescent phenotype.

These results evidencing the contribution of a PTGS2 dependent-PGE_2_ pathway in the senescence of human epidermal keratinocytes are similar to those we previously obtained with human dermal fibroblasts undergoing replicative senescence [37]. Therefore, PGE_2_ could be a universal player in senescence, whatever the senescence inducer -oxidative stress in keratinocytes, telomeres shortening in fibroblasts- and whatever the cell type. However, the involved EP receptor type differs in the two contexts, EP3 in fibroblasts *versus* EP4 in keratinocytes. Both are high affinity receptors for PGE_2_, but they transduce the signal through different G-proteins, Gi for EP3 and Gαs for EP4, with a downstream canonical signaling pathway involving the phospholipase C or the PI3K respectively [48].

It is now clearly established that accumulation of senescent cells in tissues and organs [2, 49] plays a causal role in the effects of aging, by limiting the organismal lifespan and increasing the incidence of many age-related disorders and pathologies [50–52]. Interestingly, the inhibition of PTGS2 was shown to extend the life span in C. Elegans [53], highlighting the crucial role of PTGS2 in aging. To support this, aging was shown to be associated with a severalfold increase in PTGS expression, production of PGH_2_, and downstream synthesis of active prostanoids [54, 55]. For example, serum from 24-month-old rats showed significant increase of PGE_2_, compared to 6-month-old rats [56]. Similarly, PGE_2_ levels from elderly persons were higher than those in young ones [57]. Our present results show that the PTGS2 protein level was higher in the epidermis of elderly subjects than in young ones support and enlarge these data, highlighting a general role for prostaglandins in aging.

To decipher the molecular mechanisms that lead to transformation in a context of aging, we took advantages of our cultured NHEK model. Unlike to NHDFs which are stably and irreversibly cell cycle arrested when they reach an *in vitro* replicative senescence plateau associated with shortened telomeres, NHEKs prematurely enter *in vitro* senescence well before reaching a critical telomere length, because of an increase in endogenous oxidative stress. Again, in contrast to the replicative senescent state of NHDFs, the senescent state of NHEKs is unstable, with the vast majority of cells that die by autophagic programmed cell death, and a very few of them that spontaneously and systematically escape cell death and re-enter in the cell cycle to generate post-senescent cells harbouring preneoplastic and neoplastic markers [18, 22, 21]. Thereby, this model could mimic the physiopathology of the earliest carcinogenesis step and give access to a mechanistic understanding of this step, and not just to the pre-transformed state once established. In previous studies, we had identified several parameters controlling the ability of senescent NHEKs to re-enter cell cycle: The metabolism of xenobiotics by cytochrome p450 pathway via AKR1C2 and AKR1C3 (also known as Prostaglandin F synthase) that probably enhances the fitness of the senescent cells [21]; the level of ER stress that has to be resolved [41]; and the levels of oxidative stress and autophagy -which are strictly linked- which have to be not too high (unless lethal) but much higher than those of exponentially growing cells [19]. Here, we show that invalidation of PTGS2, its pharmacological inhibition, or the inhibition of the EP4 receptor decreased the frequency of neoplastic escape, strongly suggesting that the PTGS2/PGE_2_/EP4 axis is another parameter associated with the early steps of epithelial cell transformation. Therefore, the selective inhibition of PTGS2 or EP4 could be a therapeutic avenue to make regress the preneoplastic lesions that often comprise a high percentage of senescent cells [58, 59], or block their malignant evolution that could operate by senescence evasion. In support to this proposal, it should be noted that high PTGS2 expression was found in actinic keratoses, the pre-neoplastic lesions precursor of squamous cell carcinoma [60, 61]. Interestingly, diclofenac (an inhibitor of PGE_2_ synthesis among its mechanism of action) is currently used for the topical treatment of actinic keratoses, with good tolerance and very good efficacy [62–65]. In addition, it was reported that the homozygous deficiency of either PTGS1 or PTGS2 reduces skin tumorigenesis by 75% in a multistage mouse skin model [39].

More generally, there are evidence of a role of the PTGS2/PGE_2_ axis in causing and promoting the development of a variety of cancers, including non-melanoma skin cancers (basal cell carcinoma and squamous cell carcinoma) and melanoma [66–69]. Other evidences suggest that EP1, -2, and -4, but not -3, play a role in tumorigenesis [48]. Our results indicate that EP4 would be the principal EP receptor by which PGE_2_ exerts its effects on NHEK senescence. This is of importance because EP4 has been shown to be a tumor promoting receptor in skin, and in particular, can increase melanoma proliferation and metastasis [70]. In contrast, the inhibition of the EP4 receptor has been shown to inhibit tumor growth, angiogenesis, lymph angiogenesis, and metastasis [71].

In conclusion, we report that in epidermal keratinocytes, the PTGS2/PGE_2_/EP4 pathway plays a regulatory role in senescence, and regulates the mechanism of pre-transformation by senescence evasion, depending on its level of activation. Moreover, our findings confirm that prostaglandins are components of the SASP [35, 38, 72], possibly contributing to its inflammatory and protumorigenic effect together with cytokines, growth factors and MMPs already described. These results may provide a foundation for future anti-aging and anti-cancer therapeutic approaches. Since carcinomas often develop in the context of advanced age, a specific pharmacological inhibition of the PGE_2_ pathway might represent a useful strategy to, at the same time, eliminate the senescent pre-cancerous cells of pre-neoplastic lesions such as actinic keratosis, prevent their switch from senescence to pre-transformation, and reduce the global inflammatory context of aging that favors the growth of cancer cells.

## MATERIALS AND METHODS

### Reagents and cell culture

Normal human epidermal keratinocytes (NHEKs) were purchased from Lonza (Basel, Switzerland), Clonetics (CC-2501), or Promocell (Heidelberg, Germany). We used cells from 6 different donors from various ethnic origin and age (referred as 4F0315, K3MC1, K23FC1, K40FH1, K67FA1, KNBMC1). Cells were obtained anonymously and informed consent of each skin donor was obtained by the supplier. Cells were grown at 37°C in an atmosphere of 5% CO_2_ in a KGM-2 BulletKit medium consisting of modified MCBD153 with 0,15 mmol/L calcium, supplemented with bovine pituitary extract, epidermal growth factor, insulin, hydrocortisone, transferrin, and epinephrin (Clonetics). Such a serum-free low-calcium medium was shown to minimize keratinocyte terminal differentiation [73]. In all experiments, cells were seeded at 3500 cells/cm^2^ and always splitted at 70% confluence. The number of population doublings (PDs) was calculated at each passage by means of the following equation: PD = log (number of collected cells/number of plated cells)/log2. The exponential growth phase is defined as the phase during which cells divide regularly, do not display the enlarged and spread morphology nor express the SA-β- Gal marker. Depending on the donor, the senescent stage was reached after a slightly different number of population doublings. NHEKs were considered senescent when having reached a growth plateau during which they display increased cell size, polynucleation, accumulation of vacuoles and various damaged components, and SA-β-Gal activity, as described in [18]. Emergent cells resulted from an atypical budding mitosis generating clones of PSNE cells as described in [22].

NS398, rofecoxib, PGE_2_, were obtained from Sigma (St Louis, MO), EP1 antagonist (AH6809, dissolved in DMSO), EP4 antagonist (L-161,982, dissolved in DMSO), EP1 agonist (iloprost, dissolved in DMSO), EP4 agonists (L-902,688, dissolved in ethanol; Rivenprost, dissolved in methyl acetate) were purchased from Cayman Chemicals (Ann Arbor, MI, USA).

### Prostanoid competitive assays

Young and senescent NHEKs transfected by siRNAs or exposed to the indicated drugs were seeded at 20,000 cells per well in 6-well plates. The culture medium was changed every day. PGE_2_, PGD_2_ and PGF_2_α levels in the supernatant medium were assayed at day 4 after transfection by using respectively a prostaglandin E2 Kit (RPN222, Amersham Cytiva), prostaglandin D2 kit (E4718-100 BioVision), prostaglandinn F_2_α kit (Abcam, ab133056), according to manufacturer's instructions. The concentration of PGE_2_, PGD_2_ and PGF_2_α measured in the culture medium corresponds to the PGE_2_, PGD_2_ and PGF_2_α secreted and accumulated in the culture medium during 24 h. Once seeded, the NHEKs are recounted when collecting the conditioned media. Then, the conditioned medium of the exponentially growing NHEKs is diluted with complete culture medium, in order to have equivalent numbers of cells between the different conditions. Finally, a given volume is used for the competitive assays. The values obtained are expressed in pg/mL and therefore come from the same number of secreting NHEKs whatever the conditions.

### Immunofluorescence on cells and tissue sections

NHEKs were fixed with 4% paraformaldehyde (PFA) in PBS and permeabilized with 0.2% Triton-X-100. Slides were incubated with the primary antibody diluted in the presence of 5% BSA. They were then washed three times with PBS and incubated with the secondary antibody diluted in PBS with 0.5% casein. After three washes, nuclei were stained with Hoechst 33258 at 1 µg/mL for 3 min. Primary antibodies used were: mouse anti-PTGS2 (Santa Cruz sc-7951, 1:250) and anti-XRCC1 (Abcam, ab1838, 1:500). Secondary antibodies were IgG conjugated with rhodamine or FITC, purchased from Jackson laboratories. Human skin samples were obtained from the SkinAge Project (NCT02553954) at Oscar Lambret Centre (Lille, France), anatomopathology department, according to the French regulations. Skin punch biopsies of 5 mm were obtained from healthy young (18–40 years) and older (55 years and more) subjects (see Supplementary Table 1). The study was approved by local (CPP Nord Ouest) and national (ANSM) ethics committee and subjects gave informed written consent prior to inclusion in this study. Biopsies were embedded with OCT into a plastic cryomolds before freezing. After frozen sectioning (6 µm) on a microtome-cryostat, sections were mounted onto slides for analysis. Sections were fixed in PFA 4% for 10 min, and washed in PBS. Non-specific binding was blocked by incubation in 5% bovine serum albumin in PBS. Primary antibody was incubated for 1 h at 37°C or overnight at 4°C. The used antibodies are: anti-PTGS2 (Santa Cruz, sc-7951, 1:50), anti-XRCC1 (Abcam, ab1838, 1:200), anti-EP1 (Cayman Chemical, 101740, 1:500), and anti-EP4 (Cayman Chemical, 101775, 1:400). After washings in PBS, tissue sections were incubated with AlexaFluor 488 anti-IgG Rabbit (A21206; Molecular Probes) or AlexaFluor 488 anti-IgG Mouse (A-21202, Molecular Probes) for 60 min at RT. Finally, tissue sections were washed in PBS, nuclei were stained for 5 min with DAPI (Roche, Life Science Products, Basel, Switzerland) at 1 µg/mL and mounted in Glycergel (Dako). Optical sectioning images were taken using LSM 780 Confocal Microscope (Zeiss, Germany). AxioVision or ZEN software (Zeiss) were used for microscope image analysis.

### Epidermis fluorescence quantification

The mean PTGS2 fluorescence of each skin layer was measured using ImageJ. The boundaries of each cell in the basal layer (BL) were delimited using the drawing/selection tools of ImageJ. The « area integrated density » and the « mean grey value » were established for each cell. Then, the corrected total cell fluorescence (CTCF) was calculated using the formula CTCF = Integrated Density – (Area of selected cell X Mean fluorescence of background). The mean fluorescence of the background was obtained by selecting multiple regions with no fluorescence then calculating the average level of the grey value. A minimum of 25 cells per layer were selected individually in order to obtain a mean fluorescence per layer.

### Cytokine array and ELISA

To prepare NHEKs-conditioned medium (NHEK-CM), we plated a total of 2 × 10^6^ cells onto 100 mm plates. Twenty-four hours later, the complete culture medium was replaced with 10 mL basal KGM2 medium. After another 24 hours, the supernatant was aspirated gently, filtered through a 0.22 µm filter, transferred to ultrafiltration conical tubes (Amicon Ultra-15 with membranes selective for 3 kDa), and centrifuged to concentrate the NHEK-CM. Control medium (KGM2) was generated in the same way except there were no cells in the plate. Cytokine array assay (Proteome Profiler Human Cytokine Array, R&D systems, ARY005B, Minneapolis, MN, USA) was performed as described by the manufacturer. IL-10 (D1000B, R&D Systems, Minneapolis, MN, USA), GM-CSF (Abcam, ab174448) and G-CSF (Abcam, ab188390) ELISA assays were performed according to the manufacturer’s instructions. Once seeded, the NHEKs are recounted when collecting the conditioned media. Then, the conditioned medium of the exponentially growing NHEKs is diluted with complete culture medium, in order to have equivalent numbers of cells between the different conditions. Finally, a given volume is used for the ELISA assays. The values obtained are expressed in pg/mL and therefore come from the same number of secreting NHEKs whatever the conditions.

### RNA isolation, reverse transcription, PCR and quantitative real-time PCRs (qRT-PCR)

Total RNAs were extracted using RNeasy mini-columns (QIAGEN, Venlo, Netherlands). One µg of RNA was reverse-transcribed using random hexamers, Superscript III and dNTPs (Life Technologies, Carlsbad, NM, USA) in a final volume of 20 µl according to manufacturer’s instructions. Quantitative real-time PCR reactions were performed using the Mx3005P Real-time PCR system (Stratagen, Kirkland, WA, USA). Primer’s sequences are listed below.

**Table:** 

**Name**	**FWD**	**REV**
PTGES1	5′-GAGTAGACGAAGCCCAGGAA-3′	5′-AGTATTGCAGGAGCGACCC-3′
PTGES2	5′-CTGCAGAAGGGACACGTCTT-3′	5′-AGGACCTCCACGCAGAGC-3′
PTGS2	5′-CAAATCCTTGCTGTTCCCACCCAT-3′	5′-GTGCACTGTGTTTGGAGTGGGTTT-3′
PTGDS	5′-CCTACTCCGTGTCAGTGGTG-3′	5′-TCCGTCATGCACTTATCGGTT-3′
PGFS	5′-GAGACAAACGATGGGTGGACC-3′	5′-TGGAACTCAAAAACCTGCACG-3′
PGIS	5′-CACACCTGTGCTTGATAGCG-3′	5′-GTCGCAGGTTGAATTCTCGC-3′
TXAS1	5′-GTGATGTTTGCTTGGTTGCCT-3′	5′-CCAAGAGAGCCACTGACAGG-3′
EAR	5′-GAGGCTGAGGCAGGAGAATCG-3′	5′-GTCGCCCAGGCTGGAGTG-3′

PCR products were measured by SYBR Green fluorescence (SYBR Green Master Mix, Applied Biosystems, Waltham, MA, USA). Experiments were performed in triplicates for each data point. Results were analyzed with the MxPro software (Agilent, Santa Clara, CA, USA). Expressed Alu Repeats (EAR) was used as an endogenous control to normalize the target gene expression, and fold expression relative to the control is shown. Semi-quantitative RT-PCR were performed with primers for EP1, EP3 and EP4 that were synthesized following (Han et al., 2005). Primers for EP2 were designed using DNASTAR (Genbank N° U19487) and were as follow: EP2 forward 5′-GCGCATCTCTTTTCCAGGCACCCCACCAT-3′, EP2 reverse 5′-CATAGTCCAGCAGCGGCAGCGAGCAGAAGA-3′.

### Western-blotting

Equal numbers of young, senescent or emergent NHEKs were lysed in Laemmli buffer 2X containing SDS 4%, Glycerol 20%, Tris-HCl 50 mM pH 6.8, bromophenol blue 0.02% and 2-Mercaptoethanol 5%. Samples were denatured by heating at 95°C for 5 min. Proteins were resolved by SDS-PAGE and transferred to nitrocellulose membranes (Hybond-C extra, Amersham). Primary antibodies used were anti-human PTGS2 (Santa Cruz Biotechnology), anti-human MnSOD (Calbiochem, Darmstadt, Germany, 574596), anti-human PCNA (Dako Cytomatio, Glostrup, Denmark, M0879), anti-human GPX4 (Santa Cruz Biotechnology), anti-human PTGES1 (Santa-Cruz Biotechnology), anti-human p16^INK4^ (BD Pharmingen), and anti-human GAPDH (Chemicon International, Billerica, MA, USA). Secondary antibodies used were peroxidase-conjugated (Jackson ImmunoResearch Laboratories, West Grove, PA, USA). Peroxidase activity was revealed using ECL (enhanced chemiluminescence) or ECL advanced kits (Amersham Biosciences, Piscataway, NJ, USA).

### Small interference RNA

Invalidation experiments for PTGS2, PTGSE1, PTGDS, PGFS were performed using pools of siRNAs from Dharmacon (ON TARGET Plus Smart pool, Dharmacon, Lafayette, CO, USA). A non-targeting siRNA pool (Dharmacon) was used as control. Proliferating, senescent were plated at 70,000 cells per well in six-well plates and were transfected by using the RNAiMAX Lipofectamine reagent (Invitrogen Corp., Carlsbad, CA, USA) or by using PrimeFect siRNA Transfection Reagent (Lonza) according to manufacturer’s instructions.

### Measurement of SA-β-Gal activity

The SA-β-Gal activity was revealed as described by [37]. Briefly, Cells were fixed with 2% formaldehyde/0.2% glutaraldehyde in PBS for 10 min at room temperature. After a rapid wash with PBS, cells were incubated at 37°C overnight in the X-Gal reaction mixture (1 mg/mL X-Gal, 40 mM phosphate buffer (pH 6), 5 mM potassium ferrocyanide, 5 mM potassium ferricyanide, 150 mM NaCl, 2 mM MgCl_2_). The blue cells considered as SA-β-Gal positive were manually counted in at least five independent fields for a total of at least 100 cells for each condition.

### Measure of ROS levels

The oxidant status NHEKs was assessed using H_2_DCFDA (2′,7′-dichlorofluorescein diacetate) (D399, Molecular Probes), which diffuses across membranes and is oxidized to fluorescent DCF. Exponentially growing and senescent NHEKs were incubated with 5 µM H_2_DCFDA diluted in KGM-2 medium for 15 min at 37°C. Fluorescence was read either on adherent cells using a fluorimeter (Fluostar Optima from BMG Labtech, Ortenberg, Germany), or using a flow cytometer (Coulter EPICS XL-MCL) after trypsinization, washing, and resuspension in prewarmed PBS at 37°C.

### Calculation of PSNE frequency

After chemical treatment, senescent NHEKs were seeded into 10 cm dishes at the limit density for emergence (2,000 cells per dish). A few days later, the dishes were fixed and stained by crystal violet, the emergent clones were counted under microscopic examination, as described in [22].

### Statistical analyses

Student's one- or two-tailed *t-*tests, as appropriate, were used for statistical analyses. The *p*-values are indicated in the diagrams with ^*^ for *p*-values < 0.05, ^**^ for *p*-values < 0.01 or ^***^ for *p*-values < 0.001.

## Supplementary Materials

Supplementary Figures

Supplementary Table 1
