# Supplementary material for: Prostaglandin E2 regulates senescence and post-senescence neoplastic escape in primary human keratinocytes
Source: Aging (Albany NY). 2024 Nov 18;16(21):13201–24. doi: 10.18632/aging.206149 (PMC11719115; doi:10.18632/aging.206149)
Supplement: Supplementary Figures [file aging-16-206149-s001.pdf]

SUPPLEMENTARY FIGURES

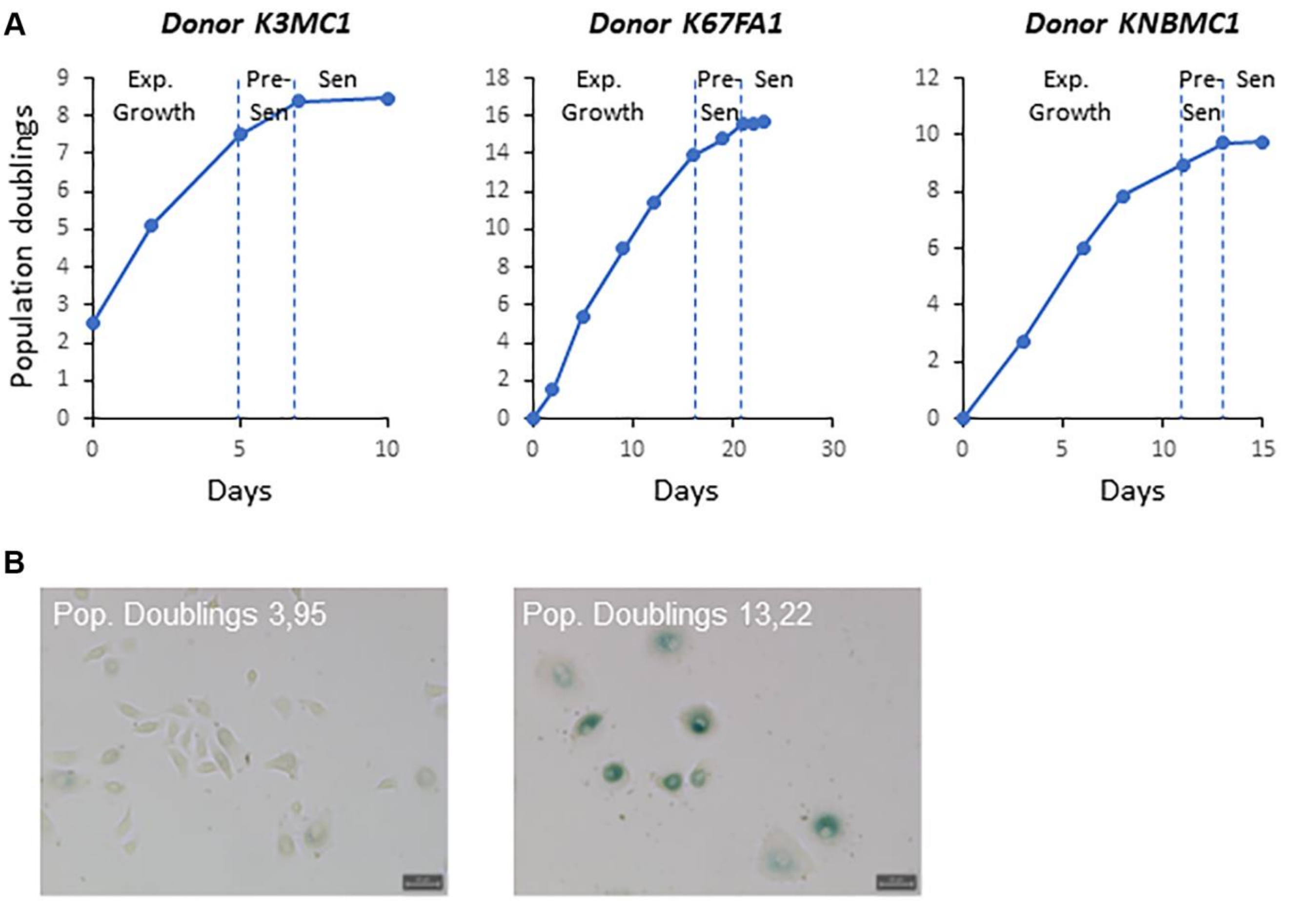

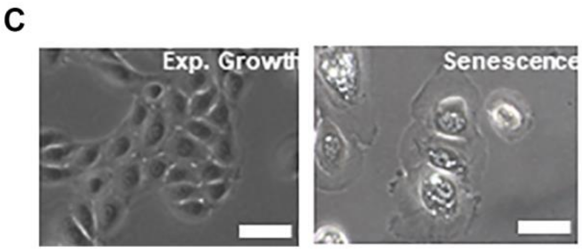

D

	FSC-A median	SSC-A median
Exp. Growth	47,02	15,50
Senescence	80,91	43,72

Supplementary Figure 1. Growth curve and senescence characteristics of *in vitro* cultured NHEKs. (**A**) Examples of growth curves of NHEKs (various donors) showing the exponential growth phase, the pre-senescent phase, and the senescence plateau. (**B**) Representative microscopy images of SA-β-Gal staining in exponentially growing and senescent NHEKs (Donor K40FH1). Bar represents 50 µm. (**C**) Cell morphologies observed by phase-contrast microscopy. The images shown illustrate the differences between proliferating and senescent NHEKs, recognizable by their large size and the presence of numerous cytoplasmic refringent or dark vesicles or particles. Bar represents 100 µm. (**D**) Flow cytometric data of NHEKs (K40FH1) at the senescence plateau of forward scatter factor (FSC-A) and side scatter factor (SSC-A).

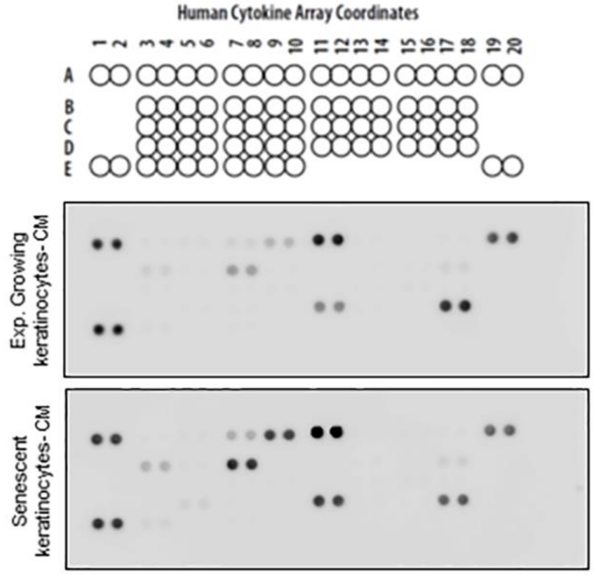

		Exp. Growing NHEKs-CM	Senescent NHEKs-CM				
Coordinate	Target/Control	Mean Intensity (a.u.)	Mean Intensity (a.u.)	Ratio (Sen/Exp.Growing)			
A3, A4	CCL1/I-309	10,7335	10,449	0,9734942			No change
A5, A6	CCL2/MCP-1	12,7225	14,199	1,11605423			Up
A7, A8	MIP-1a/MIP-1b	27,312	34,9235	1,27868702			Down
A9, A10	CCL5/RANTES	35,5275	66,9285	1,88385054			
A11, A12	CD40 Ligand/TNFSF5	96,111	100,356	1,04416768			
A13, A14	Complement Component C5/C5a	10,743	12,534	1,16671321			
A15, A16	CXCL1/GROa	9,6085	12,764	1,32840714			
A17, A18	CXCL10/IP-10	10,1925	11,113	1,0903115			
B3, B4	CXCL11/I-TAC	20,518	32,3985	1,57902817			
B5, B6	CXCL12/SDF-1	11,397	13,457	1,18074932			
B7, B8	G-CSF	58,651	89,4215	1,52463726			
B9, B10	GM-CSF	10,2805	16,3835	1,59364817			
B11, B12	ICAM-1/CD54	9,915	11,4535	1,15516894			
B13, B14	IFN-g	9,9505	11,401	1,14577157			
B15, B16	IL-1a/IL-1F1	10,335	12,7075	1,22955975			
B17, B18	IL-1b/IL-1F2	18,4475	21,4725	1,16397886			
C3, C4	IL-1ra/IL-1F3	10,522	12,904	1,22638282			
C5, C6	IL-2	9,79	11,3335	1,15766088			
C7, C8	IL-4	11,0315	12,934	1,17246068			
C9, C10	IL-5	11,5685	13,3665	1,15542205			
C11, C12	IL-6	10,6025	12,431	1,17245933			
C13, C14	IL-8	10,2915	11,92	1,15823738			
C15, C16	IL-10	11,059	13,408	1,21240619			
C17, C18	IL-12 p70	10,93	11,858	1,08490393			
D3, D4	IL-13	9,8245	11,309	1,15110184			
D5, D6	IL-16	10,1265	12,077	1,19261344			
D7, D8	IL-17A	10,8955	12,867	1,18094626			
D9, D10	IL-17E	9,9755	11,387	1,14149667			
D11, D12	IL-18/IL-1F4	75,5645	89,466	1,18396866			
D13, D14	IL-21	10,593	12,0155	1,13428679			
D15, D16	IL-27	10,563	11,1275	1,05344126			
D17, D18	IL-32α	88,964	76,125	0,8556832			
E3, E4	MIF	10,8305	18,343	1,69364295			
E5, E6	Serpin E1/PAI-1	12,88	10,1245	0,78606366			
E7, E8	TNF-α	10,3445	11,739	1,13480594			
E9, E10	TREM-1	9,5195	10,749	1,12915594			

Supplementary Figure 2. SASP composition in NHEKs. Cytokine/chemokine array performed with conditioned medium from senescent and exponentially growing NHEKs. The intensity of the signals was quantified by densitometry (ImageJ) and normalized to positive controls for each sample.

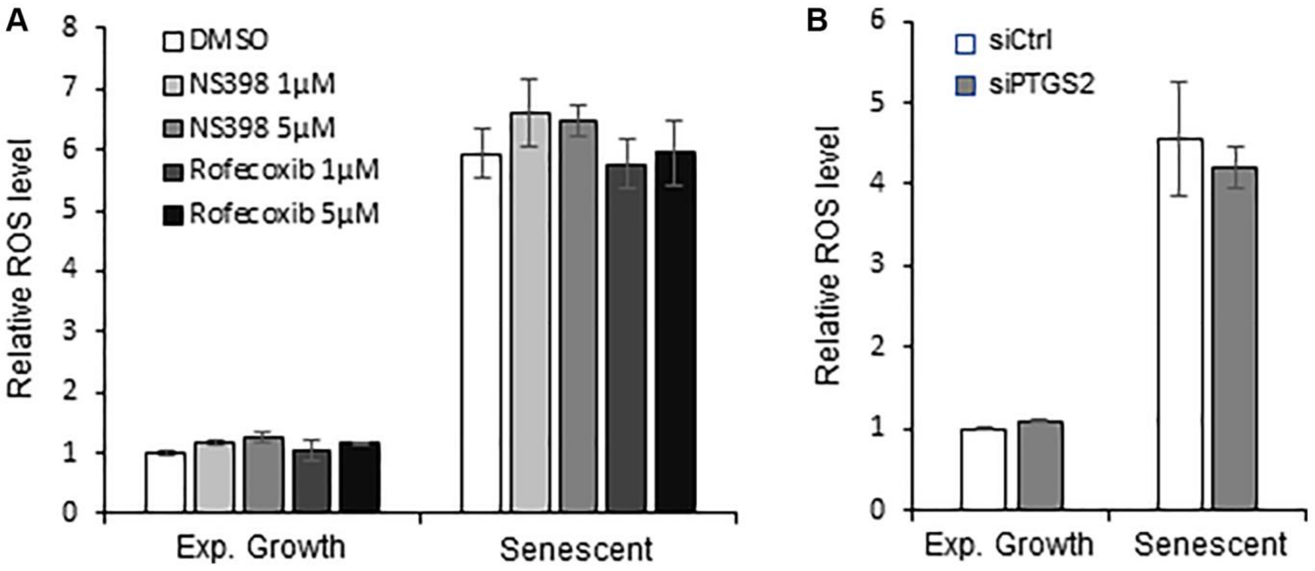

Supplementary Figure 3: Contribution of ROS-generating activity of PTGS2 to the senescent phenotype in NHEKs. (**A**) NHEKs (donor K23FC1) at the exponential growing phase or at the senescence plateau were treated or not with NS398 or rofecoxib for 16 hrs. ROS levels were analyzed by using the H_2DCFDA probe. The fluorescence was normalized to cell numbers and was expressed as relative values, a value of 1 corresponding to the level of fluorescence in exponentially growing cells. (**B**) Senescent NHEKs (donor K23FC1) were transfected with a pool of 4 siRNAs targeting PTGS2, or with non-target siRNAs. ROS levels were measured and analyzed as in (**A**). In A and B, the measures were performed in triplicate. The bars represent the mean + SD.

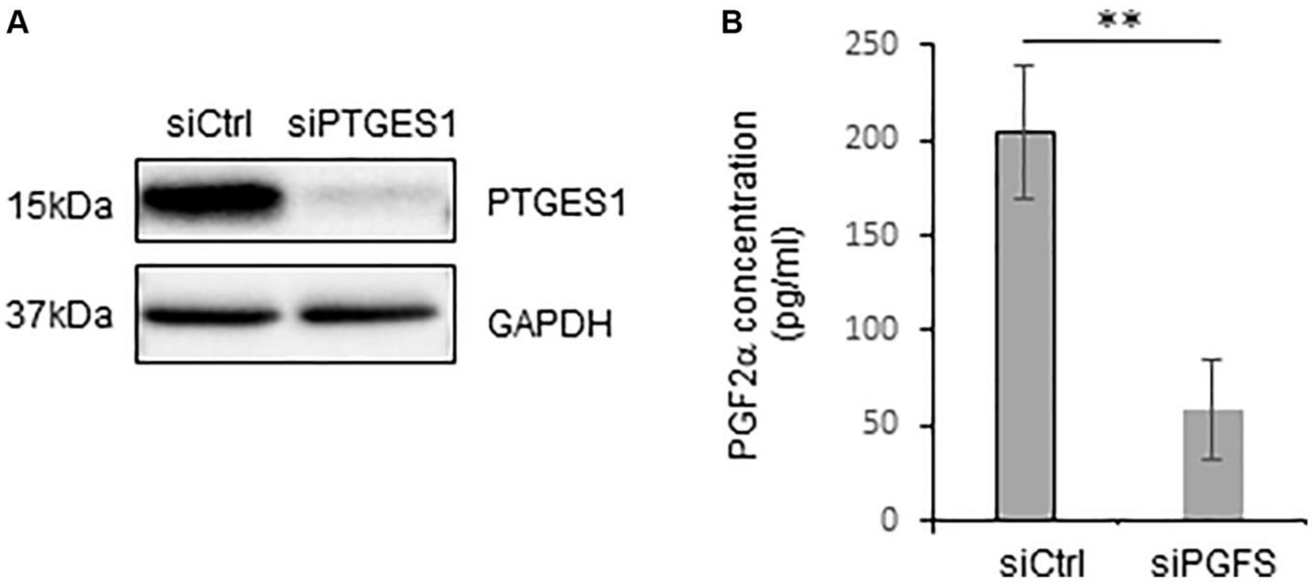

Supplementary Figure 4. Evaluation of PTGES1 and PGFS silencing efficiency. (**A**) Analysis of PTGES1 protein levels in senescent NHEKs (Donor K40FH1) subjected to PTGES1 silencing compared to a non-target siRNA as control 96h after transfection. GAPDH was used as loading control. (**B**) Analysis of the amount of PGF2α. by competitive assay in senescent NHEKs (Donor K40FH1) subjected to PGFS silencing compared to a non-target siRNA as control 96 h after transfection. The results were normalized to the number of senescent NHEKs. Bars represent the mean ± SD of three independent experiments.

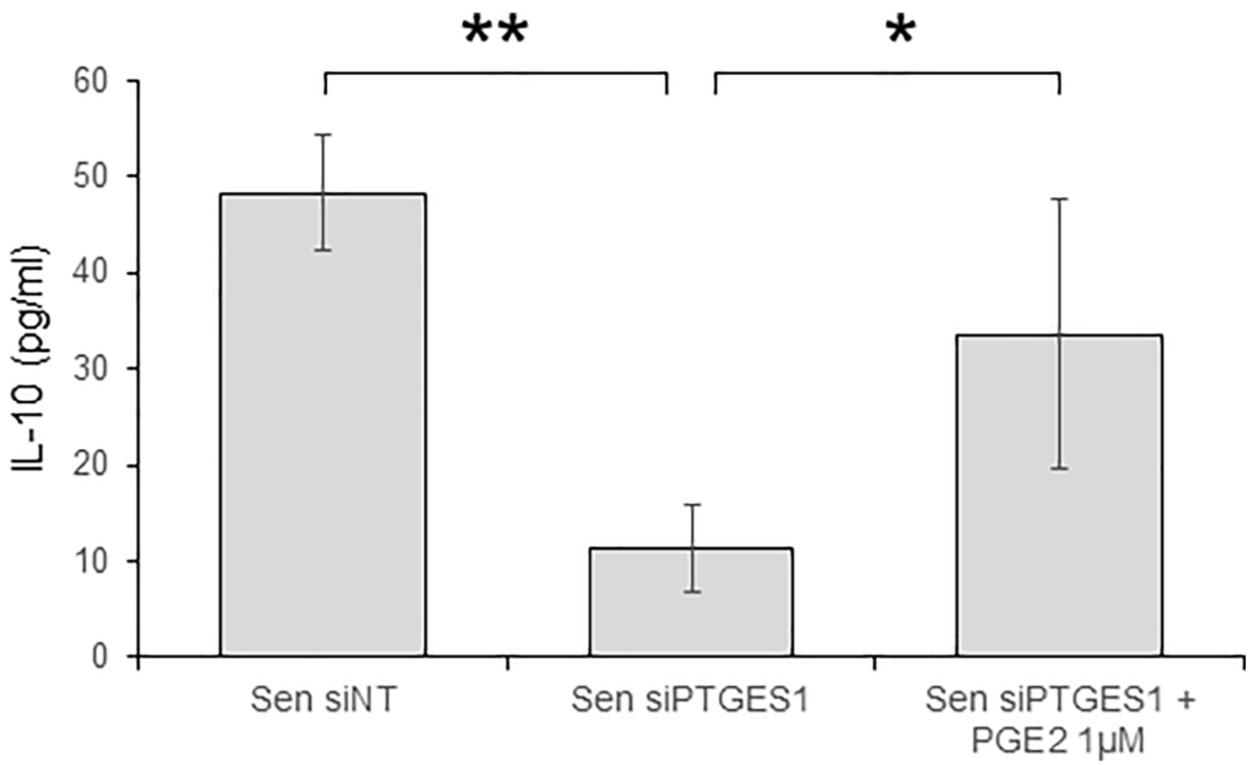

Supplementary Figure 5. PTGES1 controls the secretion of IL-10 through the production of PGE_2. Senescent NHEKs (Donor K40FH1) were transfected with a pool of 4 siRNAs targeting PTGES1, or with non-target siRNAs (siCtrl). Two days after transfection, NHEKs were treated or not with 1 µM PGE_2 4-times a day during 3 days. The amounts of IL-10 in the conditioned media (secreted) were measured by an ELISA assay. Measures were performed in triplicate. Bars represent the mean +/− SD. Significant differences are indicated with asterisks with $^{*}p < 0.05$ and $^{**}p < 0.01$.

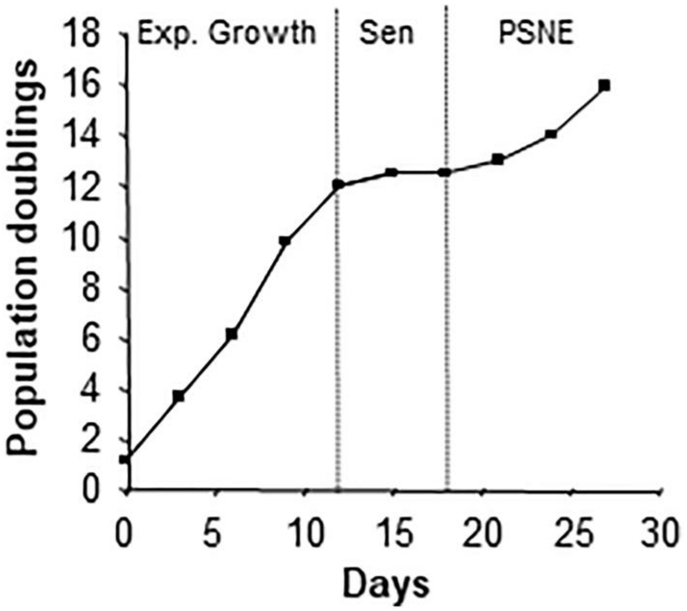

Supplementary Figure 6. Outcome of NHEKs. NHEK growth curve (from donor 4F0315) showing the exponentially growing, senescent and emergent (PSNE) phases.
