# Supplementary material for: Prostaglandin E2 regulates senescence and post-senescence neoplastic escape in primary human keratinocytes
Source: Aging (Albany NY). 2024 Nov 18;16(21):13201–24. doi: 10.18632/aging.206149 (PMC11719115; doi:10.18632/aging.206149)
Supplement: Supplementary Table 1 [file aging-16-206149-s002.pdf]

SUPPLEMENTARY TABLE

Supplementary Table 1. Characteristics of donors of skin samples.

	N° = Patient	Age	Fitzpatrick type	Localization	Smoking
Young	1	39	3	Right arm	no
	29	20	2	Right thigh	no
	46	19	3	Left hip	no
	58	39	3	Right thigh	yes
Aged	4	70	2	Right thigh	yes
	5	82	1	Left inner thigh	no
	9	87	3	Posterior right thigh	no
	55	63	3	Inner side of left knee	no
	56	79	5	Right buttock	no
